# Canonical coherence for the estimation of within- and cross-frequency cortico-kinematic interactions

**DOI:** 10.1038/s41598-026-49471-6

**Published:** 2026-05-15

**Authors:** Carmen Vidaurre, Rubén Eguinoa, Tom Maudrich, Rouven Kenville, Nerea Irastorza-Landa, Ricardo San Martín, Vadim Nikulin

**Affiliations:** 1 Basque Center on Cognition, Brain and Language, Mikeletegi Pasealekua, 69, 20009 Donostia, Gipuzkoa Spain; 2 Ikerbasque, Euskadi Pl., 48009 Bilbo, Bizkaia Spain; 3 Department of Machine Learning, TU-Berlin, Marchstraße 23, 10587 Berlin, Germany; 4 Department of Ciencias, Universidad Publica de Navarra, Av. Cataluña, s/n, 31006 Pamplona, Navarra Spain; 5 Faculty of Sports Science, Department Movement Neuroscience, Leipzig University, 04103 Leipzig, Germany; 6 Tecnalia Basque Research and Technology Alliance (BRTA), Mikeletegi Pasealekua, 2, 20009 Donostia, Gipuzkoa Spain; 7 Max Planck Institute for Human Cognitive and Brain Sciences, Stephanstraße 1A, 04103 Leipzig, Germany

**Keywords:** Computational biology and bioinformatics, Engineering, Neuroscience

## Abstract

Cortico-kinematic coherence (CKC) quantifies coupling between cortical activity and movement kinematics, serving as a non-invasive marker of sensorimotor integration and motor control. Conventional CKC approaches primarily assess within (linear) frequency coupling and overlook cross-frequency interactions, which are increasingly recognized as central to corticomuscular communication. We present a novel multivariate framework that extends the canonical coherence (caCOH) method by applying a non-linear warping of peripheral measures, enabling detection of cross-frequency CKC. The method jointly analyzes multichannel EEG and acceleration signals, maximizing sensitivity to spatially distributed neural sources while accounting for frequency-specific structure. Simulations with realistic head modeling show that the approach robustly recovers underlying patterns even at very low signal-to-noise ratios, closely matching the ground truth. Application to empirical EEG and acceleration data demonstrates that cross-frequency CKC is statistically significant in most participants and interaction pairs, indicating consistent non-random coupling. We further introduce an analysis strategy to determine whether observed interactions arise from shared (e.g. due to the signal shape) or distinct cortical sources. This framework provides a multivariate tool for characterizing the neural mechanisms of motor control and offers future opportunities for investigating their disruption in neurological disorders.

## Introduction

Corticokinematic coherence (CKC) is a neurophysiological measure that quantifies the coupling between cortical activity and movement kinematics. CKC reflects the synchronization between brain signals, typically recorded via electroencephalography (EEG) or magnetoencephalography (MEG), and kinematic data such as accelerations or force measurements during voluntary or passive movements^1–3^. This coupling primarily occurs at the movement frequency and its harmonics, with the strongest coherence typically observed over the contralateral primary sensorimotor cortex^1,4^. CKC has emerged as a valuable tool for functional mapping of the human motor cortex, providing insights into how the brain coordinates and executes movements^2^. The utility of CKC in studying human motor control stems from its ability to capture the complex interplay between neural activity and movement execution. By analyzing CKC, researchers can investigate proprioceptive processing, sensorimotor integration, and the neural mechanisms underlying motor control^4^. Indeed, CKC provides a robust index of how effectively proprioceptive afferents are represented at the cortical level and is systematically related to gross motor performance, balance control, and age-related changes in postural stability^5–7^. The CKC magnitude predicts inter-individual differences in gross motor skills^7^, is reduced in older adults in parallel with increased postural sway^5^, and closely follows postural sway dynamics during quiet standing, with stronger coherence at 1–6 Hz reflecting more efficient closed loop cortical monitoring of center of pressure fluctuations^6^. Cross-frequency CKC—for example, between low frequency kinematics and faster cortical rhythms or their harmonics—is likely physiologically meaningful, as proprioceptive input is distributed across multiple frequency specific sensorimotor channels; such cross-frequency coupling may thus capture how the cortex multiplexes afferent signals into slow components tracking body kinematics and faster rhythms supporting predictive control and coordination^5,7^.

CKC has also proven to be valuable in clinical contexts, where differences in coupling patterns between healthy individuals and patients with movement disorders can provide insights into pathophysiological mechanisms and potential therapeutic targets^8^. For instance, studies have shown abnormal CKC in stroke patients, suggesting disruptions in neuromotor coupling that may contribute to motor impairments^8^. To date, research on CKC and corticomuscular (CMC) interactions has predominantly focused on linear relationships between peripheral measures and neural activity^9^.

Conventional methods typically analyze coherence at specific frequency bands, such as alpha, beta, or gamma, assuming direct linear coupling between cortical and peripheral signals^9,10^. Although these approaches have yielded valuable information, they fail to capture the full complexity of neural interactions that underlie motor control. Recent evidence suggests that cross-frequency neural interactions play a crucial role in corticomuscular communication^11^. These non-linear interactions enable communication between distant neural areas and integration of information across multiple spatiotemporal scales^11^. For example, studies have shown that the coupling of EEG to EMG often occurs within the same frequency band^12^, whereas the coupling of EMG to EEG more frequently spans across different frequency bands^11^. This asymmetry reflects the underlying dynamics of sensory and motor pathways, with the afferent pathway involving more synapses and thus more opportunities for non-linear transmission and cross-frequency coupling^11^. These findings contribute to an emerging notion that cross-frequency interactions are important for understanding large-scale spatio-temporal brain dynamics^13,14^.

Despite the growing significance of cross-frequency coupling in neural communication, methods for investigating such cross-frequency cortico-kinematic interactions remain underdeveloped. Current approaches are limited in their ability to detect and characterize non-linear relationships between cortical activity and movement kinematics, potentially missing crucial aspects of motor control mechanisms. Even for within-frequency cortico-kinematic interactions, the most frequently used methods are based on mass-bivariate sensor or source-space approaches.

To address this gap, we propose a novel multivariate method for extracting neural sources maximally coupled with peripheral measures across different frequency bands. Our approach builds upon the canonical Coherence (caCOH) method we previously developed^15^, which maximizes coherence between two multivariate spaces in the frequency domain. The caCOH method has demonstrated superior performance compared to traditional approaches such as Laplacian derivations in the detection of corticomuscular coherence, even under low signal-to-noise ratio conditions^15^.

In the current work, we extend caCOH by incorporating a non-linear warping of peripheral measures, enabling the detection of cross-frequency interactions between cortical activity and movement kinematics. This approach allows for the simultaneous analysis of multiple EEG channels and acceleration signals, maximizing the extraction of relevant information while accounting for the spatial distribution of neural activity. Unlike previous methods that focus on specific frequency bands or assume linear relationships, our approach can identify complex patterns of coupling distributed across spatial and frequency domains.

We validate our method through extensive simulations using realistic head modeling and demonstrate its application with empirical multichannel EEG and acceleration recordings. The simulations assess the method’s ability to accurately recover neural sources and detect cross-frequency interactions under various signal-to-noise conditions and in real experiments, where SNR is unknown. This novel multivariate approach for detecting cross-frequency cortico-kinematic coherence represents a significant advance in the field of motor neuroscience, offering new opportunities to explore the complex neural mechanisms underlying human movement and in a future step, their alterations in neurological disorders.

## Materials and methods

### Cross-frequency phase synchronization

Two univariate processes are said to exhibit cross-frequency phase synchronization when their dominant (central) frequencies $f_q$ and $f_r$ are related by a rational ratio of two integers, and their phase relationship is not uniformly distributed over time^16^. Let $\phi _q(t)$ and $\phi _r(t)$ denote the instantaneous phases of the two processes at time *t*, associated with frequencies $f_q$ and $f_r$, respectively.

Cross-frequency phase synchronization is commonly characterized by the *generalized phase difference*

1 $$\begin{aligned} \psi _{qr}(t) = q\,\phi _r(t) - r\,\phi _q(t), \end{aligned}$$

where *q* and *r* are integers that define the frequency relationship between the two signals. Intuitively, *q* and *r* specify how many phase cycles of one signal correspond to those of the other. When the frequencies satisfy the approximate relation $qf_r\approx rf_q$, a consistent phase relationship may emerge, which is reflected by a non-uniform (typically unimodal) distribution of $\psi _{qr}(t)$ over time^17^. Such a concentration indicates the presence of cross-frequency phase synchronization. In this study, we focus on the special case $f_r \approx r f_q$ which corresponds to setting $q=1$. This choice simplifies both the interpretation and the implementation of the synchronization analysis, as it directly captures scenarios in which a higher-frequency cortical process oscillates at an integer multiple of a lower-frequency peripheral signal. This situation is particularly relevant in body–brain interaction studies, where physiological rhythms often appear as harmonics of slower peripheral processes. To extend cross-frequency phase synchronization analysis to multivariate signals, we adopt the following procedure. First, peripheral signals with a frequency of interest $f_q$ are frequency-scaled so that their dominant frequency matches the cortical frequency $f_r = r f_q$. Second, cross-frequency spatial projections are computed by maximizing phase synchronization—quantified using coherence (caCOH)—between the frequency-scaled peripheral signals and EEG signals evaluated at the cortical frequency bin corresponding to $f_r$.

### Synchronization index between two univariate signals

Cross-frequency phase synchronization between two univariate signals with central frequencies $f_q$ and $f_r$, satisfying $qfr = rf_q$, was quantified using the synchronization index introduced in^17^. The analysis proceeds as follows. Each signal is first narrow-band filtered around its respective frequency of interest ($f_q$ or $f_r$). The Hilbert transform is then applied to obtain the analytic representation of each signal, from which the instantaneous phases $\phi _q(t)$ and $\phi _r(t)$ are extracted. These phases are subsequently weighted by the integers *r* and *q*, respectively, in accordance with the assumed frequency relationship, and combined to form the generalized phase difference $q\phi _r(t) - r\phi _q(t)$. The synchronization index is then computed as

2 $$\begin{aligned} k_{\textrm{sync}} = \left| \frac{1}{N} \sum _{n=1}^{N} e^{j\left( q\phi _r(n) - r\phi _q(n)\right) } \right| , \end{aligned}$$

where *N* denotes the number of time samples. The index $k_{sync}$ ranges from 0, indicating no synchronization (uniform phase difference distribution), to 1, indicating perfect phase locking. Throughout this work, the notation $f_q: f_r$ is used to denote cross-frequency synchronization, whereas $f_q: f_q$ refers to within-frequency synchronization. It is worth noting that the synchronization index is mathematically related to coherence; therefore, measuring one uniquely determines the other (see Eqs. (16)–(18) in^18^).

### Frequency scaling/warping

To estimate cross-frequency phase synchronization between peripheral (body) signals and brain signals, it is necessary to modify the frequency content of the peripheral signals while preserving their instantaneous phase structure. Specifically, the goal is to scale the frequency of a peripheral signal by an integer factor *r*, such that its phase dynamics become comparable to those of a cortical signal oscillating at frequency $f_r \approx r f_q$. Let $b_i(t), i = 1 \ldots P$, denote the peripheral signals. Their analytic representations are obtained using the Hilbert transform:

3 $$\begin{aligned} s_i(t) = b_i(t) + j \mathcal {H}\{b_i(t)\}, \end{aligned}$$

where $\mathcal {H}\{\cdot \}$ denotes the Hilbert transform and *j* is the imaginary unit. From the analytic signal, the instantaneous phase and amplitude are given by

4 $$\begin{aligned} \phi _i(t) = \arg \left( s_i(t)\right) , \qquad a_i(t) = |s_i(t)|. \end{aligned}$$

To scale the signal frequency by a factor *r*, the instantaneous phase is multiplied by *r*:

5 $$\begin{aligned} \phi _{ri}(t) = r\cdot \phi _i(t). \end{aligned}$$

Expressing the analytic signal in polar form,

6 $$\begin{aligned} s_i(t) = a_i(t)\cdot e^{j\phi _i(t)}, \end{aligned}$$

a frequency-scaled analytic signal is constructed by retaining the original amplitude $a_i(t)$ and replacing the phase with $\phi _{ri}(t)$. The corresponding time-domain signal is then obtained by taking the real part:

7 $$\begin{aligned} b_{ri}(t) = \Re \{a_i(t)\cdot e^{j r \phi _i(t)}\}. \end{aligned}$$

The resulting signal $b_{ri}(t)$ is thus a frequency-scaled version of the original peripheral signal $b_i(t)$, preserving its amplitude envelope and phase dynamics while shifting its dominant frequency by a factor of *r*.

### Canonical coherence and cross-frequency canonical coherence

Coherence quantifies the degree of synchronization between two signals and is mathematically related to phase-based synchronization indices, such as the Phase Locking Value (PLV)^18^. Consequently, maximizing coherence is equivalent to maximizing PLV. In earlier work, we introduced *caCOH*, a multivariate framework designed to maximize coherence between two datasets originating from different sources such as brain and periphery^15^. Within caCOH, the goal is to determine two linear projection vectors, $\boldsymbol{\alpha }$ and $\boldsymbol{\beta }$, associated with sets *A* and *B*, respectively. These vectors are restricted to real coefficients, under the assumption that the signals from spaces *A* and *B* undergo instantaneous linear mixing. The optimization problem can be expressed through the following objective function:

8 $$\begin{aligned} L(\boldsymbol{\alpha }, \boldsymbol{\beta }) = \frac{\left| \boldsymbol{\alpha }^{\mathsf T} {\bf C}_{AB} \boldsymbol{\beta }\right| ^{2}}{\left( \boldsymbol{\alpha }^{\mathsf T} {\bf C}_{AA} \boldsymbol{\alpha }\right) \left( \boldsymbol{\beta }^{\mathsf T} {\bf C}_{BB} \boldsymbol{\beta }\right) } \end{aligned}$$

where ${\bf C}_{AA}$ is the cross-spectral matrix of space A ${\bf C}_{BB}$ is the cross-spectral matrix of space B and ${\bf C}_{AB}$ is cross-spectral matrix between spaces A and B.

CaCOH can be applied to combinations of electrophysiological modalities, such as EEG vs. MEG, EEG vs. EMG, or MEG vs. EMG. In the present study, it is used to derive spatial filters for EEG/MEG (central signals) that are maximally coherent with peripheral measures, in particular, the acceleration recordings of the motor system. At present, the method *is restricted to processes occurring at the same frequency*, and yields pairs of spatial filters$\boldsymbol{\alpha}$ or $\boldsymbol{\beta}$ and patterns ${\bf p}_\alpha$ or ${\bf p}_\beta$ from which the source time series and spatial distributions can be reconstructed. During optimization, caCOH also estimates the optimal delay at which a specific linear mixture of channels (signals) achieves maximal coherence. In the case of CKC, where only a single acceleration signal is available, caCOH identifies spatial filters solely for EEG/MEG.

For more details, please refer to our paper^15^.

**Fig. 1:**
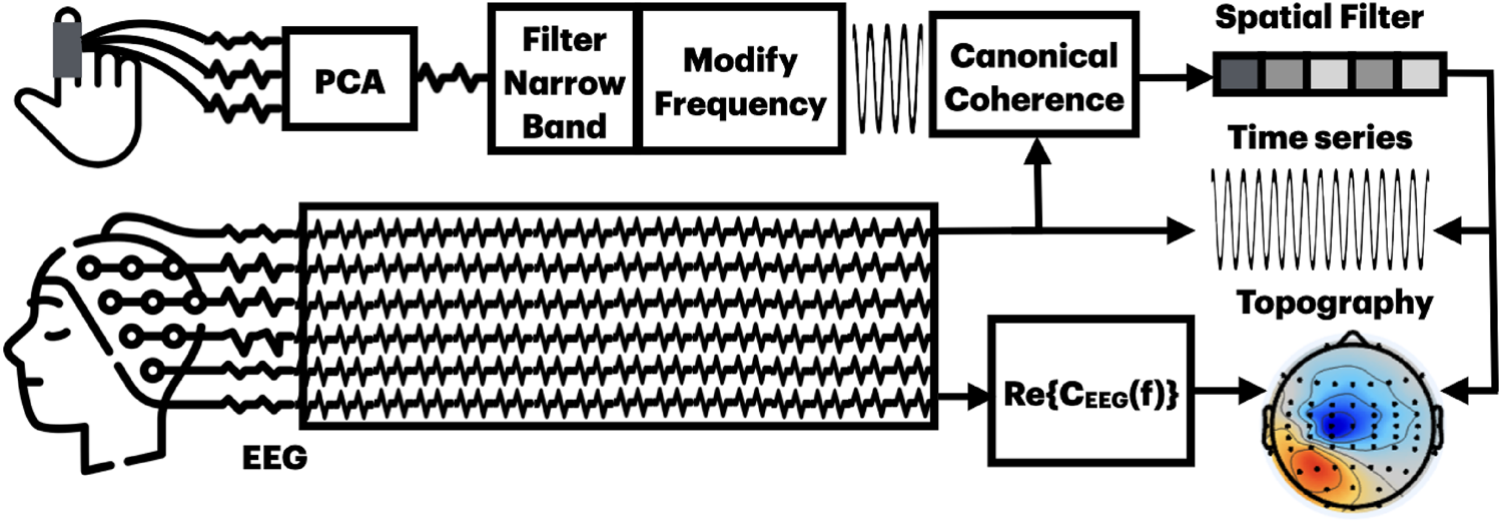
A block diagram for the main procedures of the XF-caCOH algorithm. The spatial patterns can be obtained from the filters multiplying them by the real part of the cross-spectrum of the EEG at the selected frequency bin.

In order to compute cross-frequency phase interactions we apply a frequency warping to the *slow* peripheral signal (see “ Frequency Scaling / Warping”). This is a non-linear signal manipulation that is applied to a single sensor, or to a combination of sensors mixed into one single time component, such as the first principal component of the PCA algorithm. After frequency warping canonical coherence is applied. A schematic of these procedures is presented in Fig. 1.

In this work we refer to the cross-frequency CKC interactions obtained with caCOH as XF-caCOH or XF-CKC, and the corresponding frequencies of these interactions are referred to as $f_r:f_q$. In the case of within frequency interactions obtained with caCOH, these results are mentioned as CKC strength and when we compare CKC strengths, within frequency outcomes are specified as $f_q:f_q$.

### Simulations

We performed three different simulations to test our method.

In the *first set of simulations* S1, the peripheral signals were generated like three univariate oscillatory independent time series with central frequencies 3 Hz, 6 Hz and 9 Hz independent of each other. Each oscillator was created by filtering white Gaussian noise in the frequency range the frequency band of interest using a zero-phase 4th-order Butterworth band-pass filter. The power of the oscillators decreased to the half with increasing frequency, such that the power of the 9 Hz signal was 1/4 of the power of the 3 Hz signal. The peripheral time series was obtained by summing the three oscillators together with Gaussian random noise so that the total SNR of the peripheral signal was 10, which is in the lower range of SNR values for ACC signals^19^. In the brain, five oscillatory signals were created. Two of them were cross-frequency coupled with the 3 Hz peripheral (body) oscillator: 3 Hz peripheral vs 6- and 9-Hz in the brain, i.e. 3:6 Hz and 3:9 Hz cross-frequency interactions. Each of the two cross-frequency coupled oscillators was created by warping the 3 Hz peripheral signal to 6 and 9 Hz respectively. Additionally, three other signals were independently coupled to the 3, 6 and 9 Hz peripheral oscillations (within frequency interactions at 3:3, 6:6 and 9:9 Hz). This means that there were signals with different types of couplings at the same frequency to test whether the method can extract their locations without spatial conflict between them. All signals were time-shifted twenty milliseconds with respect to the peripheral oscillators. The total simulation duration was 300 seconds, with a sampling rate of 500 Hz.

In *the second and third simulations*, S2 and S3 respectively, the peripheral and brain signals were similar and they only differed in the cortical location of the brain time series. The peripheral signals were generated like three univariate oscillatory time series with central frequencies 3 Hz, 6 Hz and 9 Hz coupled between them so that 3:6 Hz and 3:9 Hz couplings exist creating a non-sinusoidal peripheral signal (this is on contrast to S1 simulations where peripheral signals did not exhibit cross-frequency coupling 3:6 Hz and 3:9 Hz). The 3 Hz oscillator was created by filtering white Gaussian noise in the frequency range around 3 Hz using a zero-phase 4th-order Butterworth band-pass filter. To generate the oscillators at 6 and 9 Hz, the frequency of the 3 Hz signal was modified as explained in “Frequency Scaling / Warping” to be 6 or 9 Hz. The power of the oscillators decreased with increasing frequency as in the previous simulation. The time series was obtained by summing the three oscillators together with Gaussian random noise so that the total SNR of the peripheral signal was 10. Additionally, three brain oscillatory signals were created. Two of them were cross-frequency coupled with the 3 Hz peripheral oscillator: 3 Hz peripheral vs 6- and 9-Hz in the brain, i.e. 3:6 Hz and 3:9 Hz cross-frequency interactions. Each of the two cross-frequency coupled oscillators was created by warping the 3 Hz peripheral signal to 6 and 9 Hz respectively. Note that because the peripheral signal is non-sinusoidal the within-frequency couplings also exist between this signal and the brain oscillators. Additionally, one independent oscillator at 6 Hz was created in the brain, not coupled to the peripheral signal, to test whether its location could be found by our method. All signals were time-shifted twenty milliseconds with respect to the peripheral oscillator. The total simulation duration was 300 seconds, with a sampling rate of 500 Hz.

In all cases, the EEG oscillators were projected on 61 channels, arranged according to the outermost surface layer of the standard Montreal Neurological Institute (MNI) brain model^20^. The EEG forward modeling was conducted using a realistic three-shell volume conductor model^21^, incorporating the scalp, skull, and brain compartments. In the case of S1 and S2 all brain time series (5 and 4 respectively) were projected on different locations. For S3, the three brain time series coupled between each other were projected onto the same location, whereas the independent source was projected to a different location.

To model background noise, we employed 500 dipoles with uncorrelated time courses, producing noise with a 1/*f* spectral profile (pink noise). These dipoles were assigned random orientations and positions throughout the cortex.

The final brain signal was constructed by summing all oscillators together with the generated pink noise.

Phase couplings were studied using different cross-frequency factors ($r = 2, 3$) and seven brain signal-to-noise ratio (SNR) conditions for the $f_r$ components: $SNR = 0.5, 0.25, 0.1, 0.05, 0.025$ and 0.01. Each SNR was simulated 100 times leading to different locations and time-courses of the neural sources. The SNR was manipulated by adjusting the ratio of the mean variance of the projected dipole activity across channels to the mean variance of the additive 1/*f* noise generated by all background dipoles.

The recovery error or RError between a recovered ${\bf p}_{rec}$ and an original pattern ${\bf p}_{orig}$ was calculated as in^15^:

9 $$\begin{aligned} \text {RError} = 1 - \frac{|{\bf p}_{orig}^\top {\bf p}_{rec}|}{\Vert {\bf p}_{orig}\Vert \cdot \Vert {\bf p}_{rec}\Vert } \end{aligned}$$

For the simulations, the recovery error was considered a measure of performance because the optimization of XF-caCOH does not depend on spatial information. Thus, returning accurate topographies corresponding to low recovery errors implies that the method does not overfit.

#### Statistical analyses

To determine the SNR threshold at which our source localization method fails, permutation tests were performed for each simulation set across all seven SNR levels and 10 repetitions. For each repetition–SNR combination, a null distribution was generated by pseudo-randomly permuting trials in the simulations 1000 times, from which the 95th percentile was computed. The recovery error was used as the quality metric because it is independent of the optimization procedure. Performance was then quantified as the proportion of repetitions in which the recovery error exceeded the 95th percentile of the corresponding null distribution.

Additional statistical analyses were performed to assess differences in performance across the three simulation sets. We focused on the RError as quality measure because of its independence of the objective function of XF-caCOH. RError is bounded between 0 and 1, thus we used linear mixed models with a logit link function. These models are implemented instead of repeated measures ANOVA when the dependent variable is bounded, as in our case. Fixed effects included Simulation set (three levels) and Interaction (three levels for within-frequency interactions and two levels for cross-frequency results). Two SNR values, one medium high and one medium low, were selected in order to avoid 3-way interactions and models were fitted separately for each of them. A Repetition factor was included as a random effect to account for variability across realizations. To investigate specific differences between simulation sets, post hoc pairwise comparisons were conducted within each interaction level for each selected SNR, using Tukey-adjusted p-values to control for multiple comparisons within each family of tests. Statistical significance was assessed at $\alpha$ = 0.05. Odds ratio (OR) were reported to check the effect size.

### Real data

We used data acquired in the scope of another project. The study protocol received approval from the medical ethics committee of Leipzig University (approval no. 423/18-ek). All participants provided written informed consent in accordance with the Declaration of Helsinki and received financial compensation for their participation.

A total of twenty right-handed, neurologically healthy individuals (10 female, 10 male; mean age ± SD: 25.6±5.4 years) took part in the study. Handedness was determined using the Edinburgh Handedness Inventory^22^. Participants were excluded in case of neurological disorders, current pregnancy, intake of centrally acting medications, or regular engagement (>2 h per week) in organized or competitive sports involving structured training regimes, or structured musical practice. Recreational physical activity was not restricted. One participant was discarded due to the corruption of markers that impeded analyzing synchronization between brain and peripheral signals.

The participants took part in two experimental sessions separated by seven days. Each session involved a unilateral index finger tapping task with both hands at two target frequencies: 1 Hz and 3 Hz. The participants sat comfortably with their forearms resting on a cushion and were instructed to look at a cross displayed on a screen in front of them. First, participants familiarized themselves with the 1 Hz condition by synchronizing their tapping to a metronome for 20 seconds. Following this entrainment period, they performed self-paced unilateral tapping with their index finger in 12 blocks of 10 seconds each, separated by 5-second rest intervals. No metronome feedback was given during the self-paced trials. A researcher provided verbal cues indicating the start and end of each tapping and rest period. After completing all 12 blocks, the task was repeated with the opposite hand. The order of hand usage (right or left) was randomized and counterbalanced across participants. Subsequently, the same procedure was carried out for the 3 Hz condition, including metronome-guided entrainment and self-paced tapping blocks, again with randomized hand order. The entire experimental procedure was repeated on the second testing day. In this study we only analyzed data from the 3 Hz condition.

**Fig. 2:**
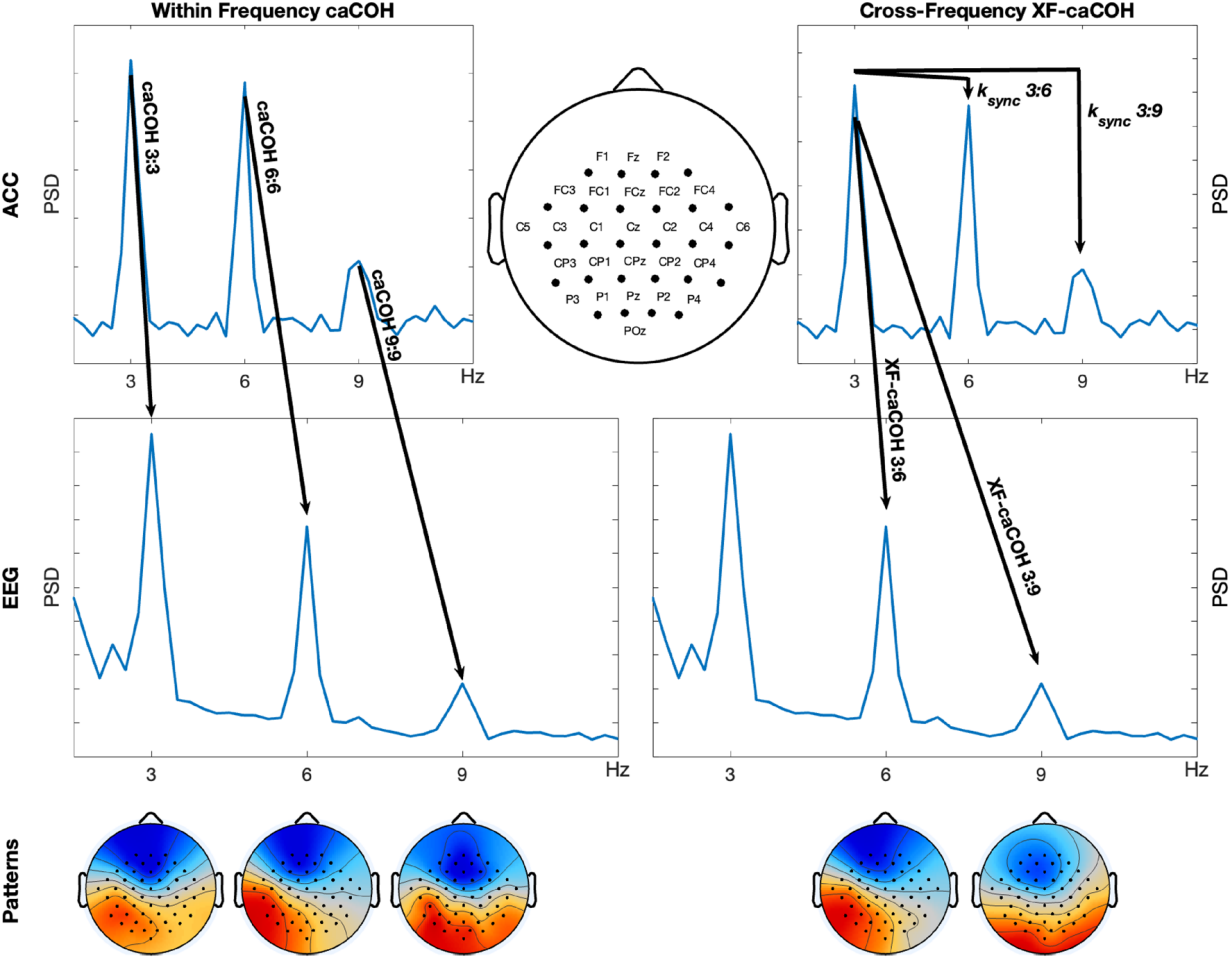
A schematic explaining within- and cross-frequency CKC interactions. Left: within-frequency interactions studied between the peripheral signal and the brain signals. For the caCOH algorithm, corresponding brain patterns are obtained. Middle: electrode layout used in the study. Right: XF-interactions computed for 3:6 and 3:9 Hz. When using only the peripheral signal, XF couplings were computed with the $k_{sync}$ at 3:6 and 3:9 Hz. The XF-interactions between periphery and brain were calculated with XF-caCOH, where the ACC was filtered at 3 Hz and its frequency shifted to 6 or to 9 Hz. Then caCOH was applied and the solutions at 6 or 9 Hz selected. Spatial patterns at the selected frequencies were also obtained.

Electroencephalographic (EEG) data were recorded using a wireless 64-channel LiveAmp system (Brain Products GmbH) equipped with active electrodes (actiCAP, Brain Products GmbH). Electrodes were individually mounted on a 128-channel cap to achieve dense coverage of bilateral sensorimotor areas. The EEG reference was placed at the left mastoid, and the ground at AFz. Electrode impedances were maintained below 5 k$\Omega$ for the duration of the recording. EEG data were continuously sampled at 500 Hz. The onset of active tapping periods was manually triggered by the experimenter, and synchronized via the amplifier’s trigger interface to ensure temporal alignment across all recorded signals. The location of the electrodes used is shown in the top row and center panel of Fig. 2. This subfigure contains a combination of points and labels, marking both all electrode positions used for the analyses.

Concurrently, electromyographic (EMG) and accelerometer data (ACC) were collected using a BrainAmp ExG amplifier (Brain Products GmbH). To ensure optimal signal quality of EMG, the skin was prepared by shaving the target area, gently abrading the surface, and disinfecting with alcohol. Monopolar recordings were obtained using sintered Ag/AgCl electrodes (4 mm diameter), positioned over the first dorsal interosseous (FDI) and abductor pollicis brevis (APB) muscles on both hands. Reference electrodes were affixed to the styloid processes of both radii, and the ground electrode was positioned on the right lateral epicondyle of the humerus. The EMG signals were sampled at 1000 Hz. ACC data were collected with two 3D acceleration sensors (Brain Products GmbH), attached with medical-grade adhesive tape to the distal phalanges of the left and right index fingers. ACC signals were acquired along the orthogonal X-, Y-, and Z-axes with a sampling frequency of 1000 Hz. Instead of using the Euclidean norm^2^ which is an only positive signal, we computed the first PCA direction of the 3D ACC signal. Thus, we retained a quasi oscillatory signal that is thought to return larger coherence results^23^. All data analyses were performed in MATLAB (v. R2023b, The MathWorks Inc.) using built-in functions, the BBCI toolbox^24^, the EEGLAB toolbox^25^ and custom functions. EEG data were cleaned using EEGLAB. First line noise frequencies were removed, and then the ’clean rawdata’ function was applied to remove noisy channels. Later, ICA was applied to automatically remove components containing eye blinks, eye movements, heart signal and muscle artifacts. The mean number of rejected components was 2.6, ranging from 1 to 6). After that, the EEG was interpolated and the BBCI toolbox applied to remove noisy trials depending on their variance^26^.

A schematic view of the interactions that are calculated using real data is presented in Fig. 2. On the left panel, one can see the within frequency caCOH interactions between ACC and EEG at frequencies 3:3, 6:6 and 9:9 Hz. At the bottom, examples of the patterns obtained with caCOH at the frequencies mentioned above. The right panel show the XF-interactions computed. XF-interactions within the first PCA component of the peripheral signal are obtained with the $k_{sync}$ index after filtering this time series around the frequencies of interest (3, 6 and 9 Hz, see “ Synchronization index between two univariate signals”). The XF-interactions between the peripheral signal and the brain signal are estimated with XF-caCOH. In particular, the peripheral signal is filtered at 3 Hz and its frequency shifted to 6 or to 9 Hz. Then caCOH is applied and the solutions at 6 or 9 Hz selected. In this case, the corresponding patterns can also be obtained.

#### Pattern similarity

The similarity between different patterns ${\bf p}_{1}$ and ${\bf p}_{2}$ obtained with caCOH and XF-caCOH in real data was measured using a similarity index $k_{sim}$ defined as follows:

10 $$\begin{aligned} k_{sim} = \frac{|{\bf p}_{1}^\top {\bf p}_{2}|}{\Vert {\bf p}_{1}\Vert \cdot \Vert {\bf p}_{2}\Vert } \end{aligned}$$

$k_{sim}$ varies from 0 (totally different patterns) to 1 (equal patterns) and serves to compare networks of sources of different interactions of interest.

#### Statistical analyses

Statistical significance of CKC and XF-CKC strength was assessed using permutation testing. For each subject and condition (left and right hand), the peripheral signal was permuted and the complete analysis pipeline was recomputed, thereby generating a null distribution corresponding to the hypothesis of temporal independence between the brain and external signals while preserving the overall characteristics of the recorded data. The observed CKC and XF-CKC values were then compared with this permutation distribution and those exceeding its 95th percentile were considered significant.

Where appropriate (for example in Table 8) p-values were corrected for multicomparison using the Holm-Bonferroni approach^27^.

Coherence values and synchronization indices between within and across frequencies ($k_{sync}$, CKC and XF-CKC) were treated as dependent variables for our statistical analyses. As they are bounded between 0 and 1, main effects and interactions were studied by fitting linear mixed models with a logit link function (beta regression for repeated measures). Fixed effects included Interaction (three level for within-frequency interactions and two levels for cross-frequency couplings) and Side (L or R). A factor “Subject” was included as a random effect to account for subject variability. Since only some main effects of the factors were significant and we did not perform multiple comparisons, no p-value adjustment was required.

### Pattern averaging

In order to compute average patterns, all topographies were first normalized (dividing by their norm). Then, they were aligned in polarity by studying the sign of the dot product between a reference pattern and all the rest of the patterns. The sign was then corrected to always obtain a positive result. After that, the average of the group of patterns was computed. Hence, cancellation effects between the topographies with opposite polarities were avoided.

## Results

### Simulations

**Fig. 3:**
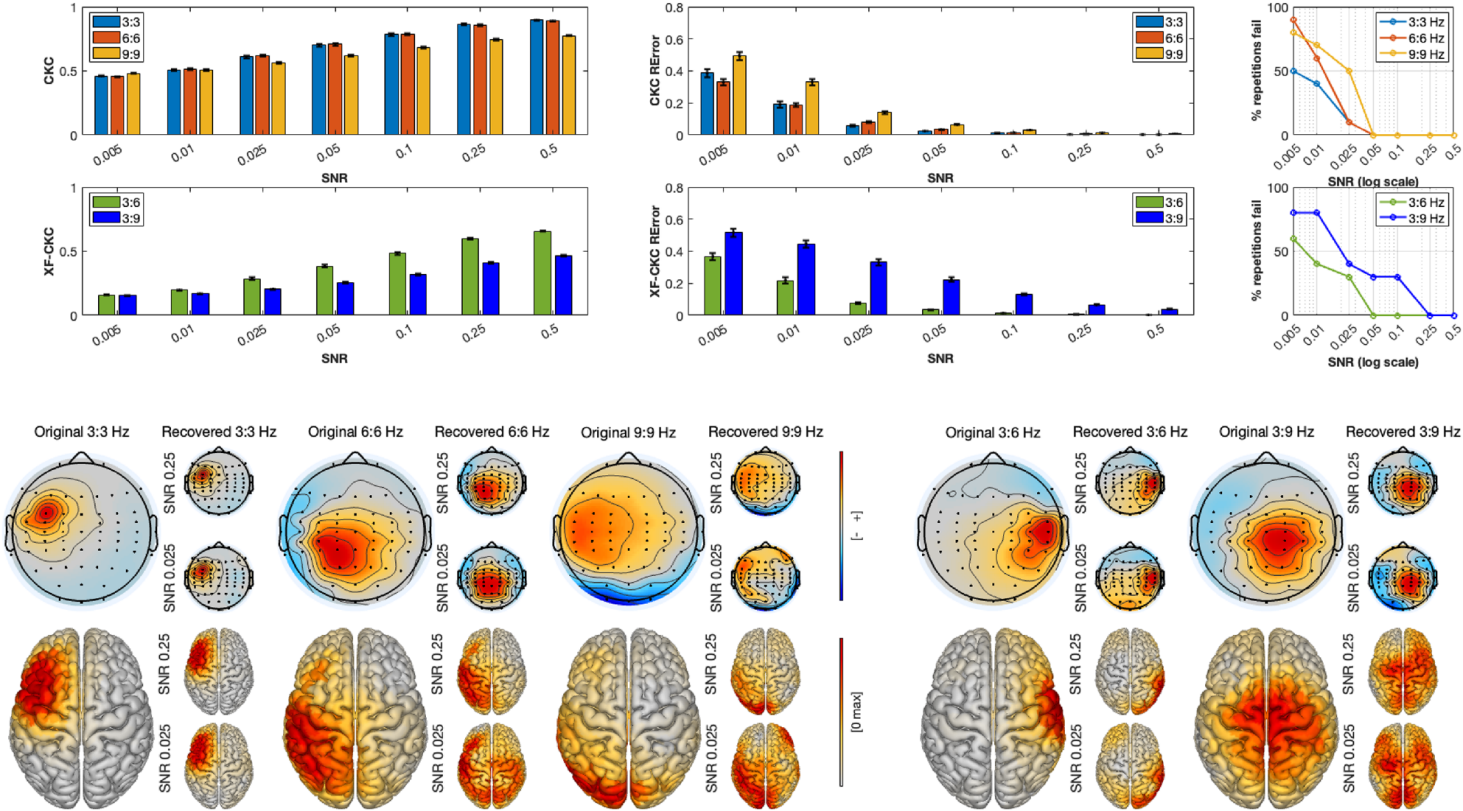
Results of simulation set 1. Top left: CKC and XF-CKC values obtained for within (3:3 Hz) and XF (3:6 and 3:9 Hz) interactions at different SNR, averaged over repetitions. Top middle: Pattern recovery errors of the same interactions averaged over repetitions. Top right: % of repetitions where the RError is pseudorandom according to the SNR. The lower panel shows the originally generated patterns at 3:3, 3:6 and 3:9 Hz together with some examples of source recovery for the same interactions at different SNRs.

**Fig. 4:**
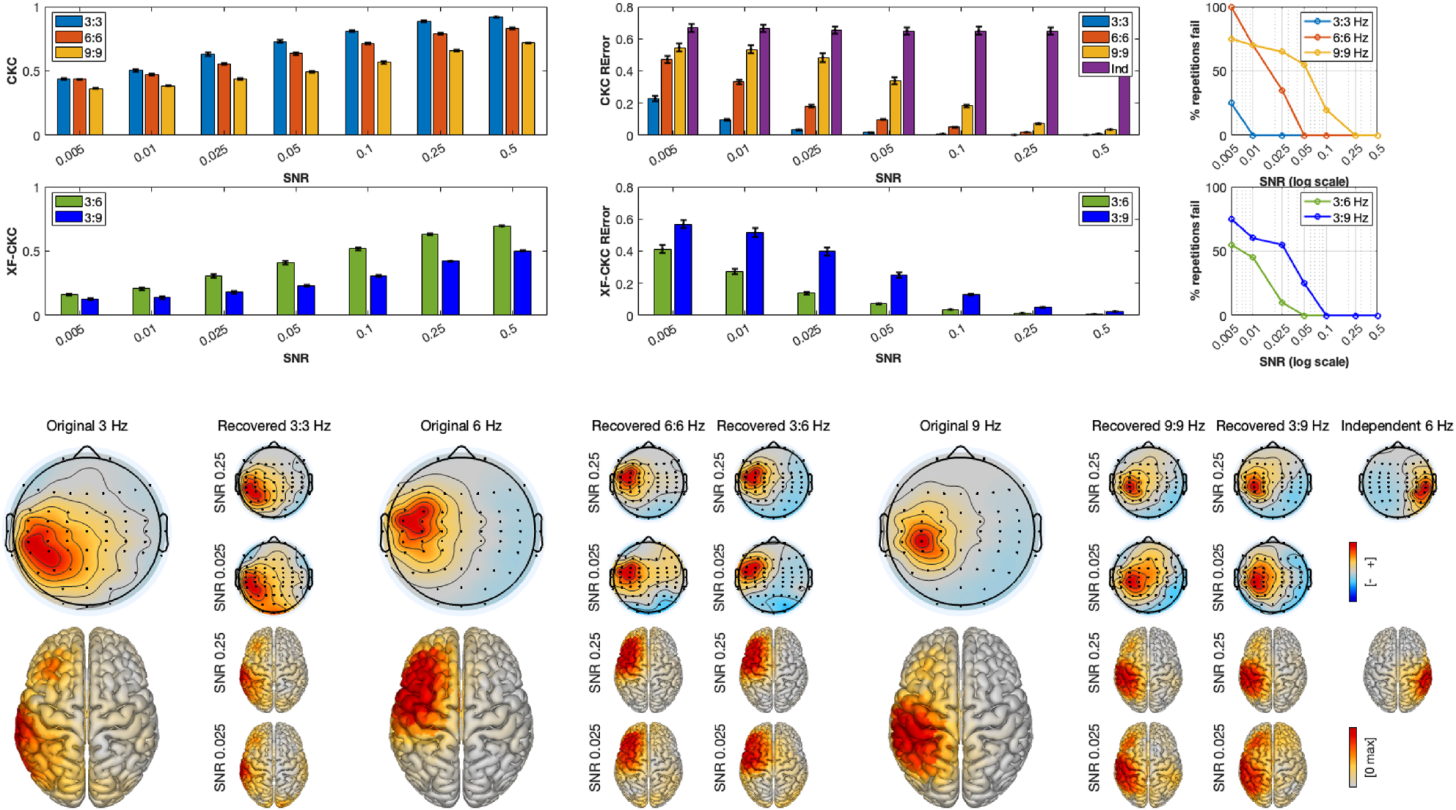
Results of simulation set 2. Top left: CKC and XF-CKC values obtained for within (3:3 Hz) and XF (3:6 and 3:9 Hz) interactions at different SNR, averaged over repetitions. Top middle: Pattern recovery errors of the same interactions averaged over repetitions. Top right: % of repetitions where the RError is pseudorandom according to the SNR. The lower panel shows the originally generated patterns at 3:3 , 3:6 and 3:9 Hz together with some examples of source recovery for the same interactions at different SNRs.

**Fig. 5:**
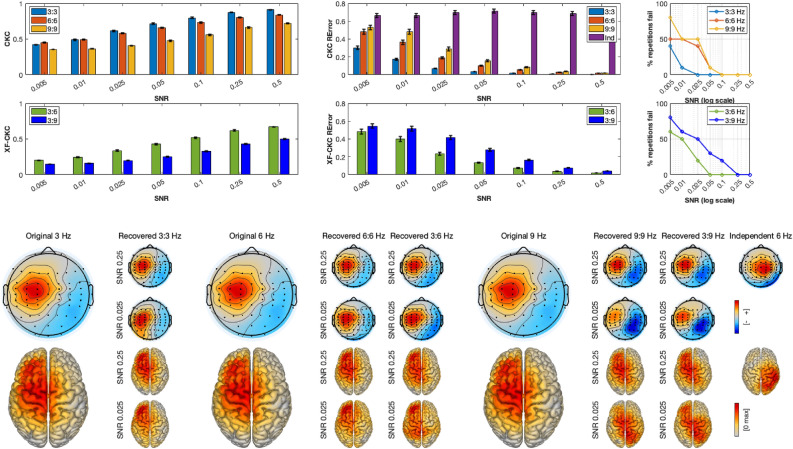
Results of simulation set 3. Top left: CKC and XF-CKC values obtained for within (3:3 Hz) and XF (3:6 and 3:9 Hz) interactions at different SNR, averaged over repetitions. Top middle: Pattern recovery errors of the same interactions averaged over repetitions. Top right: % of repetitions where the RError is pseudorandom according to the SNR. The lower panel shows the originally generated patterns at 3:3 , 3:6 and 3:9 Hz together with some examples of source recovery for the same interactions at different SNRs.

Simulation results are shown in three separate figures, one for each set of tests (Figs. 3, 4, 5). In all figures, averaged CKC and cross-frequency CKC (XF-CKC) values for each SNR are depicted in the first two rows, left corner. The error bars represent standard errors of the mean. As expected, for all cases, lower CKC and XF-CKC are recovered as the SNR decreases, with lower XF-CKC than CKC values. In the first two rows in the middle of the figures, the averaged pattern recovery errors are presented, together with the corresponding standard errors of the mean. In this case, and also as expected, a lower SNR increases the recovery error.

In the upper-right corner, the percentage of repetitions for which the RError is not significantly different from random is shown as a function of the SNR. This was computed as follows: for each repetition (i.e., source location) and interaction type (within or cross), a permuted distribution was generated by pseudo-randomly permuting trials in the simulations, repeated 1000 times for each location and SNR. The p-value was computed as the proportion of permuted errors smaller than or equal to the observed error. If the p-value was larger than 0.05, the SNR was considered to have failed at that interaction and location. The figure therefore represents the proportion of all sources for which this occurs. Across simulation sets, the method begins to lose performance at progressively higher SNRs as the frequency of the interaction increases. Cross-frequency interactions are generally weaker and fail at higher SNRs compared to within-frequency interactions. Generally, within-frequency couplings can be reliably detected down to an SNR of approximately 0.05 for lower frequencies and 0.1 for higher ones, whereas cross-frequency interactions become unreliable at around 0.1.

Examples of original and recovered spatial patterns are shown in the lower panel of Figs. 3, 4 and 5. The bigger scalp plots correspond to the originally generated patterns, whereas the small topoplots to their right are the recovered spatial patterns by caCOH and XF-caCOH at each of studied interactions: 3:3 Hz (within frequency), 3:6 and 3:9 Hz (cross-frequency) for one medium high and one medium low SNR. One can see that the recovered patterns are very similar to the simulated topographies for the higher SNR and that the recovery is less accurate with the lower SNR, specially for XF-interactions. The figures show that the algorithm works efficiently for both radial and tangential sources. In the case of Figs. 4 and 5 there is an additional column in the RError barplot corresponding to the independent source. This recovery error is very high regardless the SNR because the method cannot locate the oscillation due to the lack of interaction to the peripheral signal.

Pairwise comparisons of RError for within-frequency interactions, shown in Table 1, revealed that differences between simulation sets depended on both interaction type and SNR. At medium–high (0.25) and medium-low (0.025) SNRs, for the 3:3 Hz interaction, S1 exhibited higher error than both S2 and S3, while S2 outperformed S3. For the 6:6 and 9:9 Hz interactions, this pattern reversed, with S1 showing lower error than both S2 and S3, and S2 generally outperforming S3. Overall S1 performs better at lower SNRs than S2 and S3.

**Table 1:** Summary of pairwise comparisons of recovery error between simulations for within frequency interactions at two SNR levels (0.25 and 0.025). Values are odds ratios (OR); OR < 1 indicates lower error, p-values are in parenthesis.

SNR	Interaction	S1 vs S2	S1 vs S3	S2 vs S3
Medium–high	3:3 Hz	2.53 ($<0.001$)	1.36 (0.001)	0.54 ($<0.001$)
	6:6 Hz	0.68 ($<0.001$)	0.52 ($<0.001$)	0.77 (0.0003)
	9:9 Hz	0.28 ($<0.001$)	0.58 ($<0.001$)	2.04 ($<0.001$)
Medium–low	3:3 Hz	2.03 ($<0.001$)	1.31 (0.0713)	0.65 (0.0022)
	6:6 Hz	0.49 ($<0.001$)	0.49 ($<0.001$)	1.01 (0.998)
	9:9 Hz	0.24 ($<0.001$)	0.62 ($<0.001$)	2.59 ($<0.001$)

A similar analysis for cross-frequency interactions revealed a consistent pattern across SNR levels, extending the trends observed for within-frequency couplings. For the 3:6 Hz interaction, S1 and S2 showed lower error than S3, with differences more pronounced at medium–high SNR and slightly weaker at medium–low SNR, while S1 and S2 remained comparable. For the 3:9 interaction, S3 again tended to show higher error at medium–high SNR, but at medium–low SNR no significant differences were observed between simulations. Overall, S3 exhibited higher error where differences were present, whereas S1 and S2 performed similarly, with the effect of SNR modulating the magnitude of these differences (Table 2).

**Table 2:** Summary of pairwise comparisons of recovery error between simulations for cross-frequency interactions at two SNR levels (0.25 and 0.025). Values are odds ratios (OR); OR < 1 indicates lower error, p-values are in parenthesis.

SNR	Interaction	S1 vs S2	S1 vs S3	S2 vs S3
Medium–high	3:6 Hz	1.14 (0.0349)	0.56 ($<0.001$)	0.49 ($<0.001$)
	3:9 Hz	1.12 (0.2091)	0.79 (0.0003)	0.70 ($<0.001$)
Medium–low	3:6 Hz	1.12 (0.1460)	0.73 (0.0414)	0.66 (0.0033)
	3:9 Hz	0.99 (0.9965)	0.97 (0.9478)	0.97 (0.9706)

### Real data

#### Cross-frequency interactions on the acceleration signals

**Fig. 6:**
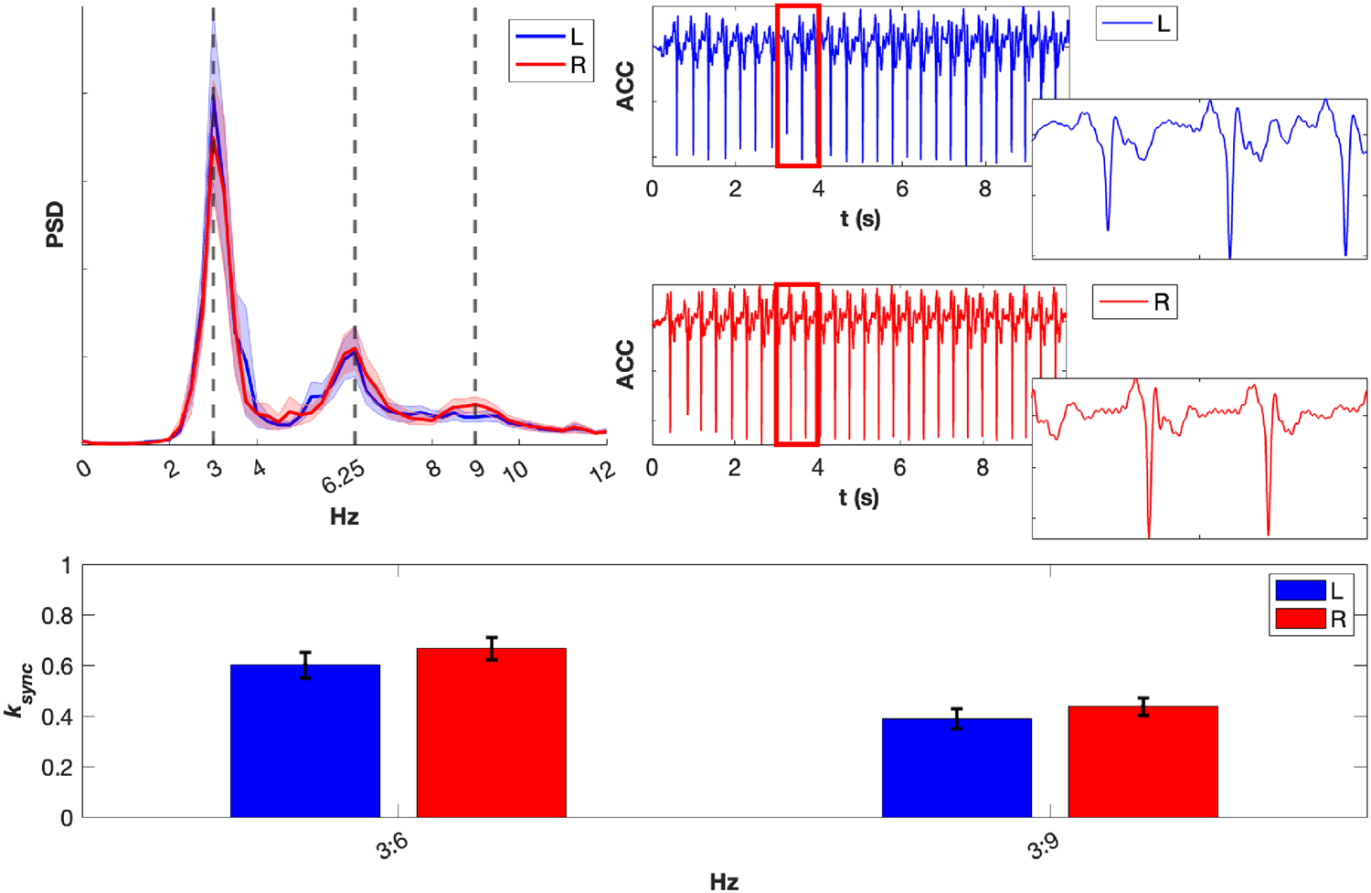
Top left: mean power spectral density (PSD) across participants for the first PCA component of the ACC signal, shown separately for the L and the R hand. The shaded area indicates the standard error of the mean (SEM). Top Right: Ten-second segment of the first PCA component of the ACC signal from a representative subject, plotted for the L hand (blue) and R hand (red). Zoomed-in panels on the right highlight the interval between 3 and 4 s in the corresponding color. Bottom: Cross-frequency synchronization index $k_{syn}$ between the first PCA component of the ACC signal at 3 Hz and the first PCA component at 6 Hz and 9 Hz (i.e. 3:6 Hz and 3:9 Hz ACC cross-frequency interactions). Blue corresponds to the L hand, red to the R hand. Error bars represent the SEM.

We studied shape, frequency content, and non-linear interaction strength of the first PCA component of the ACC signal. The two top right time series of Fig. 6 show 10 s of this ACC component for one exemplary subject. The plots show periodic signals that are not purely sinusoidal. On the right, a one-second segment of each time series is highlighted to provide a detailed view. The respective frequency content is presented on the top left panel where the spectra from all subjects were averaged. Peaks at 3 Hz and to a lesser extent 6 Hz are clear for both L and R hand. The peak at 9 Hz is clearly visible only for the right hand. In the bottom panel of the figure, one can observe an estimation of the cross-frequency synchrony between harmonics of the first PCA component of the ACC signal, for both the L and the R hand. We obtained two synchronization indices $k_{sync}$ per side (L or R), one for each interaction (3:6 and 3:9 Hz). Permutation tests performed for each subject showed that both XF interactions were significant for all subjects, except the 3:6 interaction for L hand of one subject.

Significant differences were inspected. The interaction between factors ’side’ (L or R hand) and ’XF interaction’ (3:6 and 3:9 Hz) was not significant (p-value=0.849). Thus, a simple model including only factors could explain the data. Regarding main effects, the ’side’ factor was not significant, whereas there was a very large effect of the factor ’XF interaction’, showing that the synchronization index for interaction 3:6 Hz was much larger than the one for interaction 3:9 Hz. The contrasts, odds ratios and p-values are presented in Table 3.

**Table 3:** OR and p-value for comparisons of the synchronization index $k_{sync}$ of XF interactions between ACC frequency harmonics.

	Contrast	Odds ratio (p-value)
$k_{sync}$	3:6 vs 3:9	2.53 (<0.0001***)
	L - R	0.78 (0.0823, n.s)

Signif. codes: 0.001 ’***’, 0.01 ’**’, 0.05 ’*’, 0.05 n.s.

#### Grand average CKC and XF-CKC

**Fig. 7:**
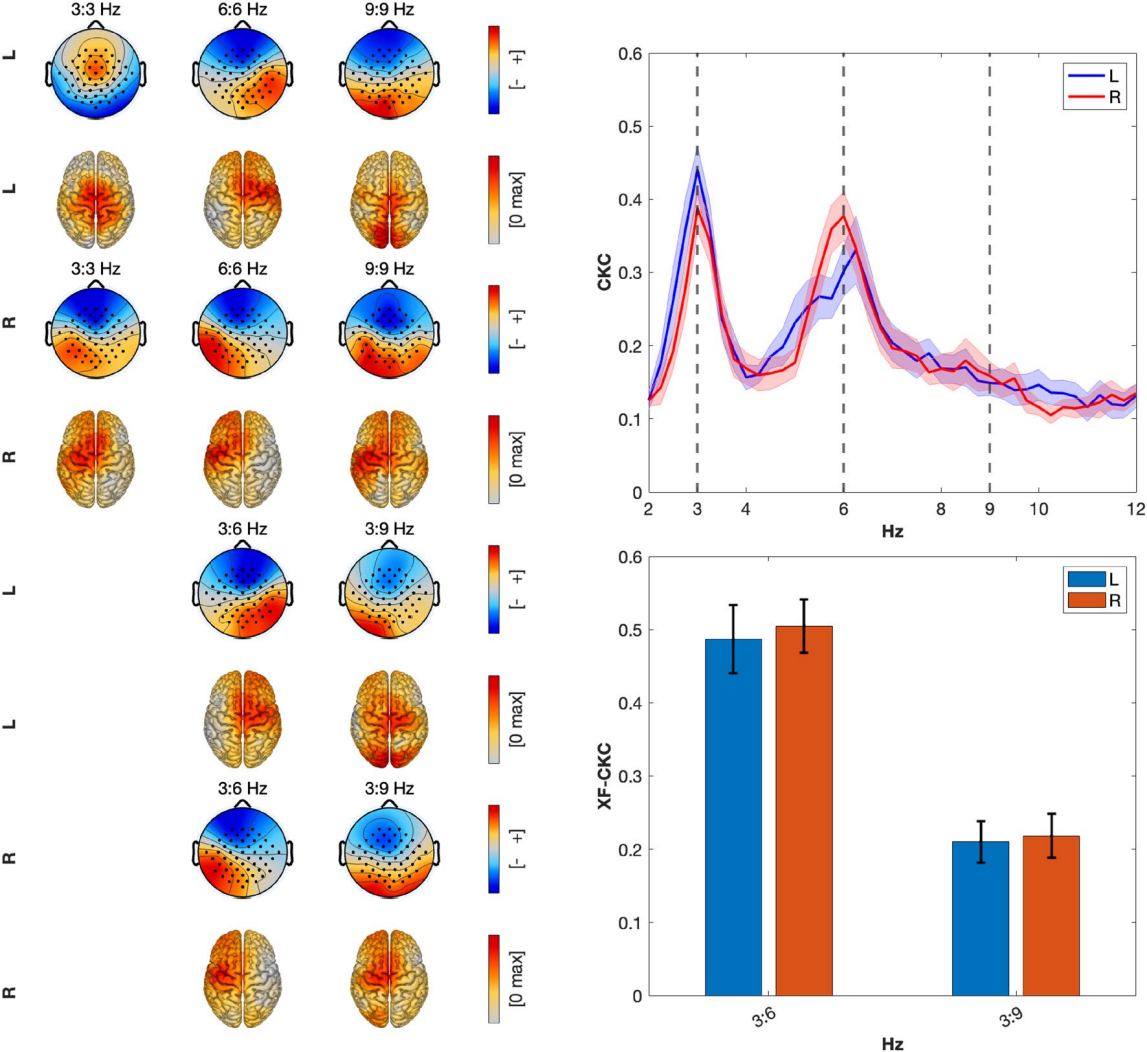
Grand average results. The upper half are caCOH results and the lower half show XF-caCOH outcomes. Left panel, columns 1 to 3: patterns and pattern localizations using eLORETA of the corresponding interactions (caCOH and XF-caCOH). Top left panel: the sources for within-frequency interactions were primarily located over sensorimotor areas including motor, somatosensory and SMA regions. One can also observe expected activation asymmetry showing stronger activity over the contralateral hemisphere, especially for the right hand. Top right panel: CKC spectrum averaged over participants. The shaded area indicates the SEM. Note prominent peaks at 3- and 6-Hz. In some subjects also 9-Hz was visible in the spectrum. The first four rows depict the results for linear caCOH results. Bottom left panel: The corresponding patterns and sources indicate sensorimotor activation with stronger activity being on the contralateral side. Bottom right panel: The strength of 3:6 Hz and 3:9 Hz cross-frequency interactions obtained with XF-caCOH.

Grand average results for real data are shown in Fig. 7. The upper half of the figure corresponds to CKC results obtained with caCOH. The lower half refers to the XF-CKC results obtained with XF-caCOH. The patterns obtained with caCOH and XF-caCOH and their corresponding localized results are visible on the left side of the figure and the CKC and XF-CKC results are on the right. In particular, the top right panel shows the CKC results obtained with caCOH over the whole frequency range. Below this graph one can find two bar plots corresponding to the XF-CKC strength of 3:6 and 3:9 Hz.

Permutation tests were used to assess the significance of each subject’s coupling strength in within- and cross-frequency interactions. Experimental values larger than the 95th percentile of the permuted results of each condition and subject were considered significant. All results were significant (all subjects and L and R hands) for CKC in frequencies 3:3 Hz, 6:6 Hz and 9:9 Hz. In relation to XF-CKC results, all 3:6 Hz interactions were significant for all subjects and both hands. On the other hand, for 3:9 Hz, 6 results were not significant for the L hand and 4 for the R hand.

Then, a linear mixed model with logit function with factors ’side’ and coupling interaction ’Freq.’ plus their interaction was fitted. The interaction was not significant (p-value = 0.096), thus a simpler additive model was used. ’Freq.’ was a significant predictor (p < 0.001), while ’side’ had no significant effect. Post-hoc tests revealed a marked drop of within-frequency CKC at 9:9 Hz compared to 3:3 Hz and 6:6 Hz, but 3:3 and 6:6 Hz were not found significantly different (see Table 4). These results suggest a frequency-dependent CKC strength where the lower SNR at 9Hz in ACC and EEG is likely driving the difference.

**Table 4:** Odds ratios for contrasts of within-frequency CKC strength values at different frequencies (Freq). Values in parentheses indicate p-values.

	Contrast (freq)	Odds ratio (p-value)
CKC	3:3–6:6 Hz	1.14 (0.450 n.s.)
	3:3–9:9 Hz	10.08 ($<0.0001$***)
	6:6–9:9 Hz	8.96 ($<0.0001$***)

Signif. codes: 0.001 ’***’, 0.01 ’**’, 0.05 ’*’, 0.05 n.s.

A similar statistical analysis was performed for XF-CKC values. Factors were ’side’ (L and R hand) and ’XF-interaction’ (3:6 and 3:9 Hz). In this case, also the best model contained only the main factors. Similarly as before, the ’XF-interaction’ was a significant predictor (p < 0.001), while ’side’ had no significant effect. Post-hoc analyses revealed a marked drop in XF-CKC strength for the 3:9 Hz interaction compared to the result at 3:6 Hz (see Table 5).

**Table 5:** Odds ratios contrasts between XF-CKC values for the two XF-interactions studied (3:6 and 3:9 Hz). Values in parentheses indicate p-values and their significance levels.

	Contrast	Odds ratio (p-value)
XF-CKC	3:6 Hz – 3:9 Hz	3.91 ($<0.0001$***)

Signif. codes: 0.001 ’***’, 0.01 ’**’, 0.05 ’*’, 0.05 n.s.

#### Origin of the interactions

In EEG/MEG, highly similar spatial patterns and strong synchronization between putatively “cross-frequency interacting” sources often point to harmonics of a single non-sinusoidal generator rather than two distinct coupled processes. Non sinusoidal waveforms inherently produce power at integer multiples of a fundamental frequency and can generate robust phase–amplitude and phase–phase cross-frequency coupling between the base rhythm and its harmonics in the absence of any true interaction between separate neural populations, as demonstrated by simulations and empirical data showing that sharp or asymmetric oscillations yield strong CFC and amplitude–amplitude coupling between harmonically related bands^14,28,29^. Thus, in an attempt to find out whether different within-frequency caCOH and XF-caCOH interactions share spatial information an analysis was performed to compare the similarity of patterns and level of synchrony between 3 Hz and 6 Hz, and 3 Hz and 9 Hz signals, within and across frequencies. The rationale is that similar EEG patterns of within (i.e. a CKC value at 6:6 Hz) and within and cross (i.e. a XF-CKC value for the 3:6 Hz) interactions would most likely indicate similar brain sources for the oscillatory processes involved in the interactions. Furthermore, if the extracted time series corresponding to those patterns are also highly synchronized, then this assumption is further supported.

The similarity of the patterns was quantified with the similarity index $k_{sim}$ computed as in Eq. (10). The average results across participants and standard errors are observed in Table 6 where the least similar patterns involving 6 Hz, are on average those of the 3:3 vs. 6:6 Hz and 3:3 vs. 3:6 Hz in the left hand. The most similar are the patterns of 6:6 Hz vs. 3:6 Hz of both the L and the R hands. Relative to the results including 9 Hz, the most similar patterns are 9:9 vs 3:9 Hz, although not to the same extent as 6:6 vs 3:6 Hz. This difference might be due to the SNR decrease between 6 and 9 Hz.

**Table 6:** Mean values with standard errors (SEM) of similarity values obtained with patterns found with caCOH and XF-caCOH. $f_q:f_q$ indicate within-frequency interactions obtained with caCOH and $f_q:f_r$ indicate cross-frequency interactions obtained with XF-caCOH.

Side	3:3 vs. 6:6 Hz	3:3 vs. 3:6 Hz	6:6 vs. 3:6 Hz	3:3 vs. 9:9 Hz	3:3 vs. 3:9 Hz	9:9 vs. 3:9 Hz
L	$0.443 \pm 0.065$	$0.484 \pm 0.068$	$0.852 \pm 0.046$	$0.402 \pm 0.060$	$0.348 \pm 0.056$	$0.559 \pm 0.086$
R	$0.622 \pm 0.070$	$0.726 \pm 0.056$	$0.884 \pm 0.053$	$0.552 \pm 0.063$	$0.513 \pm 0.059$	$0.691 \pm 0.073$

Finally, we also computed synchronization indices $k_{sync}$ between different caCOH projections. These indices quantify the degree of synchronization between two signals, with values of 1 indicating complete synchronization and 0 indicating no synchronization. In order to obtain the corresponding EEG time series, we projected the multivariate EEG onto the filter weights obtained from caCOH or XF-caCOH at the frequency bin of interest (3 Hz only for caCOH, 6 and 9 Hz for caCOH and XF-caCOH). Then, the broadband EEG was filtered in the associated band (around 3 Hz, 6 Hz or 9 Hz) and the synchronization index between different projections was estimated. For the estimation of cross-frequency interactions, the frequency of the slower signal was increased to match that of the faster signal. This in turn allowed obtaining a cross-frequency synchronization index between brain caCOH or XF-caCOH sources. The results averaged over participants are presented in Table 7.

**Table 7:** Mean values with standard errors (SEM) of synchronization index $k_{sync}$ between brain times series obtained by projecting the EEG data onto caCOH or XF-caCOH weights at the indicated frequencies.

Side	3:3 vs. 6:6 Hz	3:3 vs. 3:6 Hz	6:6 vs. 3:6 Hz	3:3 vs. 9:9 Hz	3:3 vs. 3:9 Hz	9:9 vs. 3:9 Hz
L	$0.121 \pm 0.019$	$0.134 \pm 0.018$	$0.664 \pm 0.075$	0.045 ± 0.006	0.044 ± 0.006	0.461 ± 0.068
R	$0.112 \pm 0.023$	$0.114 \pm 0.024$	$0.722 \pm 0.073$	0.060 ± 0.009	0.054 ± 0.008	0.514 ± 0.065

Table 7 shows that the synchronization between the 3:3 Hz signal and the 6:6 Hz and 3:6 Hz signals is relatively low, despite the patterns appearing fairly similar, particularly for the R hand. In contrast, synchronization between the caCOH projections at 6:6 Hz and 3:6 Hz is very high. Regarding the results involving 9 Hz, the results are similar as those at 6 Hz, meaning that the greatest synchronization is obtained between signals projected into the 9:9 Hz and the 3:9 Hz filters.

Taken together with the spatial similarity of the patterns shown in Table 6, these values suggest that only the 6:6 Hz vs. 3:6 Hz oscillations and the 9:9 Hz vs. 3:9 Hz originate from a largely overlapping network of sources and likely represent the same underlying signal, whereas the rest of sources likely represent different signals.

Finally, we assessed the correlation between $k_{sim}$ and $k_{sync}$, obtaining a total of 12 correlation values (6 per side; see Table 8). P-values were adjusted for multiple comparisons using the Holm–Bonferroni procedure^27^ to control the family-wise error rate. After correction, the correlations for 6:6 and 3:6 Hz, as well as 9:9 vs. 3:9 Hz, for both left and right hands, remained significant, further corroborating the assumption of similar underlying sources.

**Table 8:** Correlation between $k_{sym}$ and $k_{sync}$. In parenthesis adjusted p-value using Holm–Bonferroni, * indicates significant result.

Side	3:3 vs. 6:6 Hz	3:3 vs. 3:6 Hz	6:6 vs. 3:6 Hz	3:3 vs. 9:9 Hz	3:3 vs. 3:9 Hz	9:9 vs. 3:9 Hz
L	0.201 (1.000)	0.098 (1.000)	0.905($\lll$0.001*)	0.311 (1.000)	0.195 (1.000)	0.682 (0.012*)
R	0.258 (1.000)	0.089 (1.000)	0.822($\ll$0.001*)	0.318 (1.000)	0.440 (0.477)	0.758 (0.002*)

## Discussion

The present study introduces a novel methodological framework for analyzing cortico-kinematic coherence (CKC) that substantially extends previous approaches and provides new insights into the organization of sensorimotor interactions. By developing a technique based on canonical coherence, we demonstrate the feasibility of detecting both within- and cross-frequency cortico-kinematic interactions in a maximized and spatially optimized manner. This in turn enhances the interpretative scope of CKC beyond traditional sensor-based or inverse-model-dependent source analyses.

### Methodological advancements

Conventionally, CKC is studied either in sensor space, where spatial resolution is limited, or in source space, where the analysis heavily relies on biophysical forward models^30,31^. Both approaches inherently introduce confounds, either by neglecting the maximization of coherence or by depending on accurate head modeling pipelines. Over the past two decades, multivariate approaches have gained prominence in EEG/MEG analysis because they allow the extraction of spatially distributed neural patterns. These methods provide principled ways to extract networks of sources that optimize a defined objective function while rejecting components unrelated to it^15,32–34^. Our XF-caCOH framework builds on these multivariate principles by optimizing the extraction of cortico-kinematic components across all channels simultaneously, without relying on explicit forward modeling. This approach allows for a more robust and practical solution, particularly in situations of low SNR or when subtle group or condition differences are of interest. This shift toward multivariate modeling provides a richer and more robust description of sensorimotor dynamics and improves the sensitivity to subtle patterns of neural–peripheral coupling.

Indeed, our extensive simulations show that the XF-caCOH robustly recovers both within- and cross-frequency interactions across SNR levels, with performance differences between simulation sets being relatively modest. Within-frequency couplings were consistently well recovered, and even cross-frequency interactions were reliably detected at moderate SNRs. While spatial overlap of sources slightly increased error, all simulation sets yielded similar patterns of recovery, demonstrating that the method performs reliably for both distributed and co-located interactions. These results support the method’s applicability across a range of interaction types and spatial configurations.

### Spatial distribution of CKC

In agreement with previous reports^1,2,30,31^, our analyses revealed CKC sources primarily localized in sensorimotor cortices, with the strongest activations consistently observed over contralateral hemispheres. Both motor and somatosensory cortices were engaged, underscoring the bidirectional interplay between movement-related efference and proprioceptive afference. Interestingly, while the overall activation patterns for 3:3, 6:6, and 9:9 Hz interactions showed similarities, they were not completely identical. This suggests that CKC at specific frequency components is associated with spatially distinct, yet partly overlapping, cortical generators. Such a finding aligns with prior work demonstrating nuanced organization of motor output and proprioceptive input being represented in partially segregated cortical regions, thereby supporting a modular and frequency-specific organization of motor-sensory integration^35,36^. Nevertheless XF-caCOH is primarily a multivariate method for extracting pre-specified *r* : *q* phase interactions. It finds spatial patterns linked to the coupling, but their interpretation requires addressing additional questions, such as distinguishing non-sinusoidality from genuine neural cross-frequency coupling. Only in the ideal case where $k_{sim}$ is very close to 1 can one conclude that XF interactions arise from the same sources and are likely to reflect non-sinusoidal (coupled harmonics) signal structure. In practice, even when XF interactions do originate from the same source and primarily reflect non-sinusoidality, values different from 1 can also arise from noise, which slightly distorts the neural patterns. In such cases, we recommend performing a source analysis of the patterns and verifying that XF-coupled processes are associated with source configurations that show a systematic shift in source space in a specific direction. Conversely, if the sources for two patterns have a similar gravity center but mainly show stochastic fluctuations in localization, this would be more consistent with residual noise effects on the extraction of XF-coupled sources rather than distinct generators.

### Cross-frequency interactions

Another central finding of this study was the detection of robust cross-frequency coherence using the extended XF-caCOH approach. A similarity between the 3:3 Hz and 6:6 Hz CKC patterns and the 3:6 Hz cross-frequency interaction pattern was observed, yet the similarity index further demonstrated that these representations were not identical. This indicates that cross-frequency interactions reflect a distinct neural signature rather than merely harmonics of single-frequency coherence. Nevertheless, one cannot fully exclude the possibility that part of the 3:6 Hz interaction arises from non-sinusoidal contributions of peripheral signals, where movement-related sources inherently generate multiple harmonics. This issue has been extensively debated in the context of resting-state oscillations, where harmonics can spuriously contribute to cross-frequency coupling^14,29,37^ as well as in SSVEP^38^. Importantly, even if there is a relatively weak cortical source at 6 Hz that is coupled to a very strong 3 Hz cortical source (which itself is coupled to a 3 Hz peripheral signal), this strong 3 Hz generator does not prevent the detection of a coupled 6 Hz cortical source at a different spatial location in the brain, as our simulations demonstrate. These harmonically related sources are not automatically extracted from the lower frequency oscillations unless they exhibit pronounced non sinusoidal waveform properties. In summary, regardless of the exact nature of the cross-frequency interactions, our extensive simulations show that XF-caCOH is capable of reliably extracting them.

### Comparison with previous studies

Existing studies have demonstrated the presence of cross-frequency corticomuscular interactions and their relevance in both health and disease. Yet there were no studies showing cross-frequency interactions for CKC. For example, Xie et al.^39^ showed that cross-frequency coupling between alpha and gamma muscle-cortical interactions differed between healthy individuals and stroke patients, while Guo et al.^40^ reported reduced coupling from low-frequency EMG to beta EEG in dystonia patients using cross-frequency transfer entropy. Although informative, these approaches were limited to sensor-based analyses and lacked optimal spatial filtering, potentially obscuring the true strength and localization of interactions. By contrast, the XF-caCOH method directly maximizes coherence and thereby enhances detection sensitivity. Moreover, coherence as a measure is grounded in phase synchronization^18^, providing a more direct neurophysiological interpretation than methods based on transfer entropy, which conflate amplitude and phase contributions and complicate mechanistic insight. Thus, XF-caCOH presents a more physiologically transparent and computationally efficient tool for studying both within- and cross-frequency coupling.

### Limitations, applications and future directions

A methodological limitation of XF-caCOH is that the choice of the frequency-warping integers require prior knowledge or a hypothesis about the frequency pairs of interest. An important direction for future work would be to extend the framework to enable data driven discovery of the most significant coupling ratios by finding pairs of significant XF-caCOH strength. Also, since our method is designed primarily to extract coupled components, additional analyses may be needed to distinguish genuine from spurious interactions. However, we believe these analyses, which have been discussed in previous studies^29,41^, lie beyond the scope of the present work.

Despite this, XF-caCOH opens several promising avenues of application. First, the extracted CKC components can be directly compared across experimental conditions, as well as across groups of participants and patients. This feature is particularly advantageous for clinical research, where subtle differences in corticomuscular or cortico-kinematic interactions may serve as biomarkers of neurological dysfunction. Second, the identified components lend themselves to practical applications, including real-time monitoring and neurofeedback. Previous studies on corticomuscular coherence have demonstrated the feasibility of neurofeedback based on phase-synchronous activity^42^. Complementary evidence from sensorimotor functional connectivity shows that cortico-cortical coupling is related to motor imagery performance^43^, indicating that connectivity patterns–both between cortical areas and between cortex and muscles–encode behaviorally meaningful information that can be shaped through training. By analogy, CKC-based neurofeedback–facilitated through XF-caCOH could potentially allow training of movement-related cortical activations and proprioceptive integration, and might create in the future novel opportunities for motor rehabilitation in clinical populations^44^.

In summary, the proposed caCOH framework significantly enhances the analysis of cortico-kinematic interactions by enabling the optimized detection of within- and cross-frequency couplings without reliance on forward models. Our findings confirm known cortical generators of CKC while revealing frequency-specific distinctions in their spatial signatures. Moreover, the ability to capture cross-frequency coherence establishes XF-caCOH as both a relevant research tool and a promising avenue for applied neurotechnology.

## Data Availability

Participants’ raw canonical coherence and cross-frequency canonical coherence values are available in the supporting material. Supporting codes to compute canonical coherence and signal warping are available at https://github.com/CarmenVidaurre/cacoh For further inquiries regarding the data or code, please contact one of the corresponding authors.
